# Selecting a Brief Cognitive Screening Test Based on Patient Profile: It Is Never Too Early to Start

**DOI:** 10.3390/jcm13196009

**Published:** 2024-10-09

**Authors:** Gemma García-Lluch, Ariadna Muedra-Moreno, Mar García-Zamora, Beatriz Gómez, Rafael Sánchez-Roy, Lucrecia Moreno

**Affiliations:** 1 Cátedra DeCo MICOF-CEU UCH, Universidad Cardenal Herrera-CEU, 46115 Valencia, Spain; gemma.garcialluch@uchceu.es (G.G.-L.); mar.garcia1@alumnos.uchceu.es (M.G.-Z.); 2 Department of Pharmacy, Universidad Cardenal Herrera-CEU, CEU Universities, 46115 Valencia, Spain; 3 Research Group in Alzheimer’s Disease, Instituto de Investigación Sanitaria La Fe, Avda. Fernando Abril Martorell, 106, 46026 Valencia, Spain; 4 Denia Health Department, Jávea Health Center, 03700 Alicante, Spain; muedra_ari@gva.es; 5 Department of Health, Manises Hospital, 46940 Valencia, Spain; gomez_beatrizgar@gva.es; 6 Neurology Service, Arnau de Vilanova Hospital, 46015 Valencia, Spain; sanchez_rafroy@gva.es

**Keywords:** cardiovascular risk, cognitive impairment, cognitive screening, neuropsychiatric disorder, anticholinergic burden

## Abstract

**Introduction**: Cognitive impairment, marked by a decline in memory and attention, is frequently underdiagnosed, complicating effective management. Cardiovascular risk factors (CVR) and anticholinergic burden (ACB) are significant contributors to dementia risk, with ACB often stemming from medications prescribed for neuropsychiatric disorders. This study evaluates cognitive profiles through three brief cognitive tests, analyzing the impact of CVR and ACB presence. **Methods**: This cross-sectional study was performed between 2019 and 2023 in community pharmacies and an outpatient clinic in Valencia, Spain. Eligible participants were patients with subjective memory complaints 50 years or older with clinical records of cardiovascular factors. Patients with conflicting information regarding diabetes diagnosis or not taking concomitant medications were excluded. Three brief cognitive tests (Memory Impairment Screening (MIS), Semantic Verbal Fluency Test, and SPMSQ) were assessed. CVR was calculated using the European SCORE2 table, and ACB was assessed using the CALS scale. **Results**: Among 172 patients with memory complaints and CVR factors, 60% failed at least one cognitive test. These patients were on significantly more medications and had higher blood pressure and HbA1c levels. An increase in CVR and ACB was associated with more failed tests. Additionally, elevated SCORE2 scores were associated with a greater failure rate on the MIS test, while patients with elevated ACB more frequently failed the SPMSQ test. **Conclusions**: Selecting an adequate brief cognitive test according to patients’ characteristics offers an opportunity to screen patients who are probably cognitively impaired. Whereas the MIS test may be helpful for patients with cardiovascular risk, SPMSQ stands out among patients with significant ACB.

## 1. Introduction

Cognitive impairment (CI) is a state of cognition characterized by a decline in cognitive functions such as memory, attention, and problem-solving. It is often associated with neuropsychiatric disorders and leads to the loss of functional capacity, which eventually affects daily life activities [1]. The estimated global prevalence of this disease is 50 million people, and given the increase in life expectancy, it is expected to triple by 2050 [2].

Subjective memory complaints (SMC) are characterized by subtle cognitive changes and is one of the first CI symptoms. They refer to concerns expressed by individuals who experience cognitive symptoms or difficulties despite showing no evident impairment on objective psychometric tests [3]. Patients with suspected SMC may eventually progress to CI. When this occurs, they often reach a specialist in the advanced stages of the disease or with a significant degree of CI, resulting in a high rate of underdiagnosis, especially in the early stages [4]. This situation reduces therapeutic and clinical research opportunities and underscores the urgent need for action. Hence, CI represents a pressing global challenge at the healthcare and socio-economic levels [2], emphasizing prevention and early diagnosis as the main current challenges.

The American Heart Association defines “optimal brain health in adults” as the absence of smoking, regular physical activity, adherence to a healthy diet, and a body mass index of less than 25 kg/m [4]. Three ideal health factors are defined: untreated blood pressure below 120/80 mmHg, untreated total cholesterol below 200 mg/dL, and fasting blood glucose below 100 mg/dL [5]. It has been observed that patients with higher cardiovascular risk (CVR) are more likely to have altered biomarkers associated with neurodegeneration [6]. Epidemiological studies, such as the Baltimore Longitudinal Study of Aging [7], show a significant increase in the likelihood of dementia in the presence of a cerebral infarction, regardless of infarction symptomatology. Importantly, there is compelling evidence that controlling CVR factors can significantly delay the progression of the disease [8]. The latest 2024 statistical update from the American Heart Association highlights a significant link between CVR factors and the growing global burden of brain diseases [9]. Therefore, addressing CVR factors presents an opportunity for the prevention of cardiovascular disease and dementia, given the common underlying mechanisms.

Psychiatric symptoms, such as behavioral disturbances or anxiety, are often associated with cognitive impairment. Frequently, medications with anticholinergic properties are prescribed to treat these conditions, making their use chronic. Anticholinergic burden (ACB), resulting from the cumulative effect of one or more drugs with anticholinergic properties, can cause peripheral effects, such as dry mouth or blurred vision, and central adverse reactions, like confusion or cognitive impairment [10]. Some studies have linked a high ACB with an increased risk of ischemic stroke [11,12]. Additionally, previous research indicates that patients with higher ACB tend to have lower cognitive scores [10] and that those who also have heart disease, such as coronary artery disease, perform worse on neuropsychological tests [13]. Nevertheless, little is known about the cognitive status of patients with concomitant cardiovascular factors and elevated ACB.

The present study aims to determine whether populations with higher CVR and significant ACB are more susceptible to CI than patients with these factors altered independently. Additionally, this study evaluates which brief cognitive test is most effective in detecting CI based on the patient’s profile to optimize early detection and improve translational interventions.

## 2. Materials and Methods

The study’s data are part of the Cátedra DeCo project, which aims to identify people with SMC and possible cognitive impairment through screenings in community pharmacies. Once patients are identified, they are referred to a physician for further evaluation.

We considered patients to have SMC if they reported frequent forgetfulness in recent months, their family noticed memory loss, their pharmacist observed that they forgot things, patients had trouble counting when paying, or made repeated visits for items they had already picked up, among other symptoms.

For this cross-sectional study, clinical and demographic data were collected contemporaneously with the screenings conducted between January 2019 and December 2023 through patient interviews in community pharmacies [14].

### 2.1. Inclusion and Exclusion Criteria

Eligible patients were those 50 or older with SMC who provided informed consent and did not have a previous diagnosis of Alzheimer’s Disease (AD) or other dementias, mental illnesses, or other sensory deficits, resulting in a sample of 668 patients.

**Figure 1:**
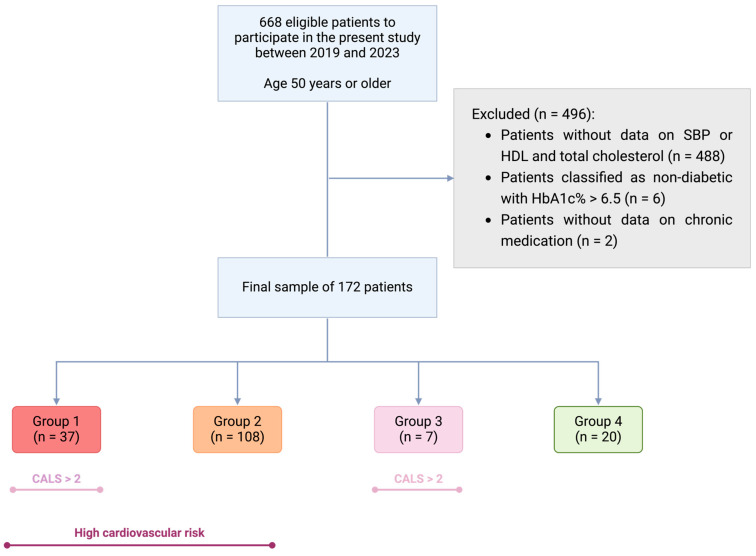
Participant selection diagram for this study. Created in BioRender. San Pablo CEU, F. (2024) BioRender.com. Group 1: High cardiovascular risk (CVR) and anticholinergic burden (ACB) ≥ 3 (red); Group 2: High CVR and ACB < 3 (orange); Group 3: No CVR and ACB ≥ 3 (purple); Group 4: No significant CVR and ACB < 3 (green, reference group). Dark purple indicates high CVR; light purple indicates ACB < 3, according to the CALS scale. Abbreviations: ACB: Anticholinergic Burden; HDL: High-Density Lipoprotein Cholesterol; CVR: Cardiovascular Risk; SBP: Systolic Blood Pressure.

For the present study, exclusion criteria included patients without available information on cardiovascular factors necessary to calculate the SCORE2 score, such as systolic blood pressure (SBP), non-HDL cholesterol and smoking habit (*n* = 488); conflicting information regarding the diagnosis of diabetes (patients without a diabetes diagnosis but with hemoglobin A1C (HbA1c) levels > 6.5% (*n* = 6); and patients without data on chronic concomitant medication (*n* = 2) (Figure 1).

### 2.2. Cognitive Status Assessment

Following the recommendations of the Conselleria de Sanitat de la Comunitat Valenciana [15], three neuropsychological tests were administered to detect patients with possible CI. The patients were evaluated using Memory Impairment Screening (MIS) [16], Semantic Verbal Fluency (SVF) [17], and Pfeiffer’s Short Portable Mental State Questionnaire (SPMSQ) [18]. These sensitivity, specificity, and duration of these tests are shown in Table S1.

The MIS was chosen because it assesses verbal learning through the reading and subsequent free and facilitated recall of four words. Scores range from 0 to 8, depending on whether the four words are recalled independently or with facilitated assistance [16]. We considered patients with possible CI when the score was ≤4 points [16].

The SPMSQ is valid for illiterate populations and is widely used in primary care due to its simplicity [19]. It scores based on the number of errors from 10 items, with coding errors scored as “1” and correct responses scored as “0”. The items include orientation tasks (“What is today’s date?”), memory tasks (“What was your mother’s maiden name?”), and attention tasks (“Subtract three from 20 and keep subtracting three from each new number until the end”). Individual cognitive scores range from 0 to 10 errors, with lower values indicating better cognitive performance. A score ≥ 3 indicates possible CI [18].

Finally, the SVF test asks subjects to name words belonging to a semantic category (e.g., animals) within a limited time (1 min). The SVF is widely used in neuropsychological evaluation due to its ease and quick application [20]. Patients who recalled <10 animals were considered as having possible CI [21].

Patients with at least one positive cognitive test were classified as individuals with probably CI, while those who did not fail any tests were classified as patients without CI.

### 2.3. Clinical Variables

In addition to the neuropsychological tests, various clinical and demographic variables, such as age, sex, cigarette smoking, diseases, and health conditions, were collected.

Total cholesterol, HDL, LDL, HbA1c, and preprandial glucose levels were included among the clinical variables. These values were obtained from the patient’s most recent blood test within 12 months before the interview. We asked patients to bring their last laboratory assessment results to the interview or asked for permission to review those parameters through a review of their medical records. Patients were classified as diabetic if they were taking antidiabetic medications or if records showed HbA1c > 6.4% or glucose levels over 126 mg/dL, following the American Diabetes Association criteria [22] (Figure 1). Patients with HbA1c > 6.7% without a diabetes diagnosis were excluded to avoid bias (*n* = 6). During the interview, blood pressure was measured three times at five-minute intervals, and the mean systolic (SBP) and diastolic blood pressure (DBP) were recorded. All medications taken by the patient on the day of the interview were noted, including antidiabetics, lipid-lowering drugs, antihypertensives, and anticholinergic medications. Lastly, educational level was asked during the study interview and dichotomized into high (high school, bachelor’s, master’s, or PhD degree) and low (no studies or elementary school).

CVR was calculated using the European SCORE2 tables, valid for patients between 40 and 69 years [23], and the SCORE2-OP, adapted for patients between 70 and 89 [24]. These tables, proposed by the European Society of Cardiology, calculate the 10-year risk of a cardiovascular event and are adapted and validated for different regions based on the population evaluated [23,24]. Since Spain is considered a low-risk region [23], the table adapted for populations with low CVR was used in this study. Variables used for this calculation included age, sex, smoking status, SBP, and total and HDL cholesterol levels, yielding a score between 1 and 49. Patients were categorized into low, medium, and high 10-year CVR according to the SCORE2 charts [23,24]. Patients with moderate or high CVR were classified as having “CVR Alert”, while those with mild CVR were classified as “No CVR Alert”.

Total ACB was calculated using the “CRIDECO Anticholinergic Load Scale” (CALS) [10]. This scale is based on a recent systematic review, including 217 different drugs, and classifying them, according to their anticholinergic effect, into low potency (load 1), medium (load 2), or high (load 3). Total ACB was explored continuously and dichotomously, differentiating patients without significant ACB (CALS < 3) from those with high ACB (CALS ≥ 3), following clinical guidelines [25].

All neuropsychological tests, the cardiovascular risk scale SCORE2 and SCORE2-OP, and the CRIDECO anticholinergic burden scale are publicly available in the cited references.

### 2.4. Patient Grouping

Finally, patients were categorized into four groups based on their total CVR and ACB. Group 1 included patients with moderate or high CVR and significant ACB (CVR+, ACB+); Group 2 comprised patients with moderate or high CVR but low ACB (CVR+, ACB−); Group 3 included patients with low CVR and high ACB (CVR−, ACB+); and Group 4 consisted of patients with low CVR and low ACB (reference group, CVR−, ACB−), see Figure 1 and Table S2.

### 2.5. Statistical Analysis

Descriptive analyses compared different variables between patients with CI and those without CI. Depending on whether the variable was numerical or categorical, *t*-tests and chi-square tests were used.

Logistic regressions were performed with a 95% confidence level to determine the odds ratios (OR) of developing CI based on the SCORE2 scale, groups 1–4, or the anticholinergic drug used. The reference level was Group 4 (CVR−, ACB−). Regression models were not adjusted for age or sex because those variables were not statistically significant when introduced. In secondary analyses, we adjusted by educational level (high educational level as reference group) and performed a multinomial logistic regression to compare groups 1–4 with the number of failed tests. All tests were two-tailed, and a *p*-value < 0.05 indicated statistical significance. Finally, one-way ANOVA was conducted to compare the means between test scores and (a) total CVR and (b) total ACB. Data analyses were performed using R Studio and R Commander (version 4.2.3).

### 2.6. Ethical Considerations

The study of human subjects has ethical implications. This study was reviewed and approved by the Ethical Committee for Clinical Research with Medications of the Arnau de Vilanova Health Department (MOR-ROY-2018-013). All participants signed informed consent forms to participate in the study.

## 3. Results

### 3.1. Demographic and Clinical Description of Participants

This work included 172 patients, of whom 60% (*n* = 104) failed at least one neuropsychological test. Both groups had an average age of around 74 and a similar sex distribution (60–70% women).

Among the CVR factors, a higher prevalence of CI was observed in patients with altered Hba1c% levels (6.68 ± 1.22 vs. 5.68 ± 1.12, *p*-value < 0.01) or higher SBP (Table 1). Additionally, patients with CI were taking more medications and had a significant ACB (33.65% vs. 13.24%).

Out of 172 patients, we have data regarding neurologist referral for 92, with 50 of them evaluated by a neurologist. Nine of them (18%) had subjective memory complaints, one experienced a near-syncope, thirty subjects (60%) had mild cognitive impairment, eight patients (16%) had dementia, one experienced essential tremor, and another had an unknown diagnosis. Regarding the number of failed tests among these 50 patients, 24 subjects failed one test (48%), 10 patients failed two tests (20%), and 16 subjects failed three tests (32%).

### 3.2. Increased Cardiovascular Risk and Anticholinergic Burden Are Associated with Worse Cognitive Test Performance

Despite no significant differences in the 10-year CVR and the presence of CI among the study participants (SCORE2, Table 1), we observed that higher CVR was associated with more cognitive test failures, indicating a broader impact on cognitive domains. Specifically, 89% of patients with a SCORE2 of 27.8 or higher (high CVR) failed at least one cognitive test, and this proportion reached 100% with a SCORE2 of 36.4, where all patients failed all three tests. Conversely, only 3.8% of individuals with the lowest CVR failed all tests (Figure S1A). Similarly, 86% of patients with an ACB of 3 or more failed at least one test (Figure S1B).

Next, neuropsychological tests were analyzed separately. Patients who failed the MIS test had higher SCORE2 scores (15.92 vs. 11.81, *p* < 0.01, Table 2) and a CVR Alert (moderate or high CVR according to SCORE2 [93.33% vs. 79.46%, *p* = 0.02, Table 2]). Notably, just 6.67% of patients with low CVR failed MIS. No statistically significant differences were observed between poorer scores on SVF and SPMSQ and higher CVR (Table 2).

Many patients with higher ACB failed the SPMSQ, suggesting a possible alteration of cognitive function in a different domain (Table 2). The most consumed drug classes among the study participants were benzodiazepines (*n* = 44, 39.64%), antidepressants (*n* = 31, 27.93%), and opioids (*n* = 26, 23.42%), followed by antidiabetics, primarily metformin (*n* = 23, 20.72%). Benzodiazepine use tripled the risk of CI (OR95% = 3.33 [1.53, 7.88], *p* < 0.01).

### 3.3. Patient Profile Based on Cardiovascular Risk and Anticholinergic Burden

Lastly, patients were categorized into four groups to determine whether CVR or ACB most affects the CI presence (Figure S1, Table S2).

**Figure 2:**
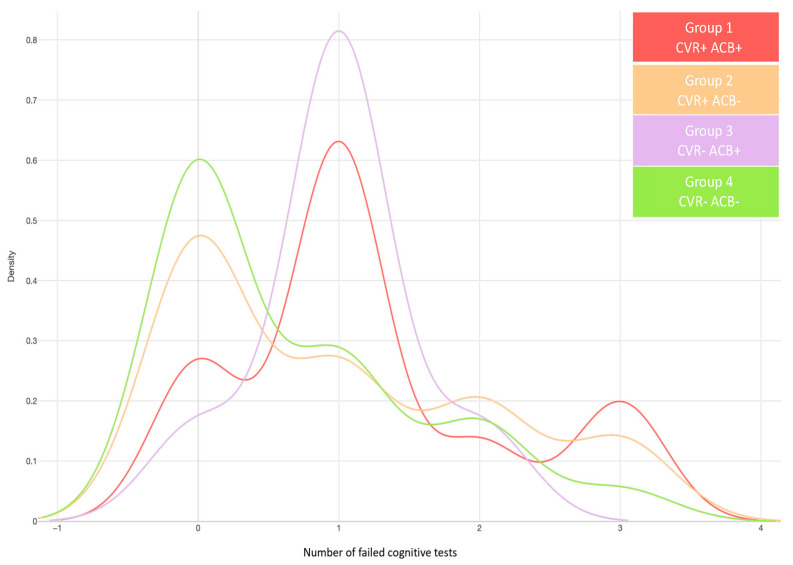
Relationship between groups and the number of failed tests. Groups 1 and 3 have more patients failing one test, with a high anticholinergic burden (ACB). Group 1 has fewer patients failing one neuropsychological test than Group 3, but more patients fail all three tests. Groups 2 and 4 have a low ACB, with Group 2 having a high cardiovascular risk (CVR). Group 1: High CVR and ACB > 2 (red); Group 2: High CVR and ACB < 3 (orange); Group 3: No CVR and ACB > 2 (purple); Group 4: No significant CVR and ACB < 3 (green, reference group).

As shown in Figure 2, patients in group CVR+ACB+ predominantly failed one test and had the highest proportion of failing all three cognitive tests. Additionally, we observed a fivefold increased risk of failing one test in this group, compared to patients without CVR and ACB (OR95%= 5.23 [1.37, 20.00], *p*-value = 0.01. Nevertheless, those differences disappeared when the educational level was added to the model.

Most patients with low CVR but high ACB (CVR−, ACB+) predominantly failed one cognitive test (Figure 2). In contrast, most patients with high CVR but low ACB (CVR+, ACB−) did not fail any tests, but they were also the second most numerous group in failing all three cognitive tests (Figure 2).

As for the odds of failing each of the tests that were assessed in this study, we observed that patients in the first and second groups (CVR+, ACB+, and CVR+, ACB−) had a higher odds of failing the MIS, suggesting a significant impact of high CVR on this test, (Table 3). More specifically, patients with both high CVR and significant ACB (CVR+, ACB+) had a fivefold increased likelihood of failing the MIS test compared to those with low CVR and ACB (CVR−, ACB−) (OR95% = 5.47 (1.31, 37.8)], *p*-value = 0.04), Table 3, and this effect seemed independent of ACB. Similarly, patients with CVR but no significant ACB had higher odds of failing this test (Table 3). Nevertheless, after adjusting the models by educational level, it was observed that the association remained just for patients with high educational level and CVR but without significant ACB (OR95%= 4.88 [1.27, 32.25], *p*-value= 0.04), Supplementary Table S3.

On the other hand, we noticed that patients with high CVR and ACB remember, on average, almost seven animals less (SVF tests) than those with low CVR and ACB, with a 95% confidence interval of −11.64 to −6.81.

Finally, although more patients with significant ACB failed the SPMSQ (Table 2), no significant differences were observed when the vascular component was studied (Table 3).

## 4. Discussion

The current study underscores the importance of assessing brief cognitive tests among older patients, especially if they have a significant CVR or ACB. Moreover, this study points out how elevated CVR increases the odds of failing the MIS test independently of ACB, while significant ACB is associated with worse SPMSQ punctuations. Finally, it underscores how concomitant CVR and significant ACB are associated with reduced executive and language functions.

Data from the Spanish National Institute of Statistics estimate that around 5.55% of the population has CI, rising to 12.36% among those over eighty [26]. Whereas the diagnosis of CI is often delayed, with symptoms manifesting for years before diagnosis, screening patients with subjective memory complaints can increase diagnosis rates by about 15% when the healthcare professional is adequately trained [15]. Given this inclusion criterion in our study, our study group shows an increase in prevalence compared to the Spanish population.

The Lancet Commission on Dementia Prevention, Intervention, and Care estimates that 45% of dementia cases could be prevented by deleting 14 modifiable risk factors [27]. This Commission established in 2020 that factors related to CVR, such as hypertension, obesity, smoking, physical inactivity, and diabetes, account for 11% of dementia cases [28]. In this sense, whereas smoking frequency seems to decline over time among the population, diabetes, obesity, and hypertension prevalence is rising [29].

Disrupted insulin signaling and its associated inflammation are potential triggers of neurodegenerative changes that lead to cognitive decline [30]. We observed higher HbA1c% levels within the CI group, showing that uncontrolled diabetic patients seem more likely to have cognitive decline. Similarly, Dove et al. concluded that poorly controlled diabetic patients have a threefold increased risk of progression of CI-no dementia to dementia [31]. Nevertheless, our results must be taken cautiously, since we did not have HbA1c% data from all the participants. Hypertension, the factor with the most considerable population-attributable fraction in most study populations [29], was also a relevant factor in our population. Specifically, higher SBP was associated with CI presence, which was also observed when we analyzed AD patients based on CSF biomarker levels in previous work [32].

Cholesterol levels were not associated with CI presence. Nonetheless, the last article from Livingston and colleagues updated cardiovascular risk factors as up to 16% of the total risk of dementia, contributing to high LDL levels with 7% of the total risk [27]. We assessed CVR using the SCORE2 scale, which includes sex and age and SBP, non-HDL cholesterol, and smoking habits, so although cholesterol did not seem significant by itself, its combination with the other CVR factors resulted in interest.

Overall, we observed that increased CVR according to the SCORE2 scale was associated with a significant prevalence of CI. Previous studies show that this scale is correlated with CSF Alzheimer’s Disease biomarkers, such as the Aß42/Aß40 ratio, and with NfL, the biomarker of neurodegeneration [6]. In our study, a higher SCORE2 was more prevalent among patients who failed the MIS test, regardless of the ACB. Interestingly, this was the only statistically significant association when the educational level was added to the models. It is important to note that, whereas the SCORE2 considers sex differences, age, SBP, and smoking habit, the MIS test correlates well with volumetric measurements of the hippocampus and entorhinal cortex, providing strong evidence for its validity in early dementia detection [16,33]. Taking it all together, patients with high CVR might have reduced volumes in these areas, leading to cognitive symptoms. Still, even though MIS is widely recognized for early dementia detection among healthcare professionals [16], further imaging studies are needed to confirm this hypothesis. Moreover, it could be of interest to explore the influence of diabetes management in further studies.

Increased drug intake is also a well-known risk factor for dementia due to the associated comorbidities and potential medication-related problems, such as interactions or adverse drug reactions, especially in older adults. Among the main adverse event drug reactions, ACB is frequently unnoticed since they are used to treat a wide range of conditions, and specific tools are needed to calculate it [34].

The most frequent manifestations of anticholinergic overload include delirium and cognitive decline [25]. These symptoms can often be confused with psychiatric conditions. As a result, they are sometimes treated with anxiolytics and antidepressants. These treatments can increase the anticholinergic burden and the risk of CI [35]. Therefore, ruling out anticholinergic overload in this type of patient is essential.

Patients with increased CVR and ACB remembered fewer animals than patients with low CVR and ACB. Low scores on the SVF test reflect language processing and production issues [36], indicating that patients with high CVR and ACB may have reduced verbal capacity and processing speed compared to patients without CVR and ACB. Similarly, previous research points out an association between poorer scores on the animal naming test and ACB among patients with coronary disease [13]. Verbal fluency tests, such as the SVF, provide valuable qualitative and quantitative insights, enriching the overall assessment process and are applicable in primary and specialized care settings [37].

Lastly, around 86% of patients with an ACB of 3 or more failed at least one test. Specifically, these patients were more likely to fail the SPMSQ. This brief test includes items related to orientation, the patient’s relationship with their environment, memory, and the ability to perform mathematical operations [38]. Additionally, the SPMSQ shows strong performance in detecting dementia in primary care settings, excelling in geriatric contexts [37]. It shares similarities with the Mini-Mental State Examination (MMSE), one of the most used and recommended tests in the Spanish National Health System guidelines. However, it was not used due to the application times, which are much longer than 5 min, and its significantly lower diagnostic performance [39]. Additionally, the MMSE does not establish specific recommendations on cut-off points or corrections and does not mention other characteristics that may also be relevant when conducting a cognitive test. A previous study assessed the effects of anticholinergic drug use and MMSE, but the results were marginally significant [40]. In contrast, patients with coronary artery disease and ACB exposure performed worse on MMSE [13], matching our results.

Cerebrovascular disease has been associated with central cholinergic dysfunction [40]. Specifically, it has been suggested that behavioral impairments associated with white matter hyperintensities may result from disruptions in cholinergic neuronal pathways [41]. On this basis, anticholinergic drugs interfere with the parasympathetic nervous system with pro-arrhythmic and pro-ischemic effects. As a result, ACB may increase oxygen requirements, enhancing the risk of ischemic events, such as strokes [34]. In this sense, an 18-year longitudinal study showed that patients with significant ACB had a 59% relative risk of incident stroke [12].

No significant differences regarding the SPMSQ test and ACB were observed when adding the vascular component. We believe this may be influenced by the absence of APOEε4 status data or the lack of stroke or ischemic events history records. A nationwide, population-based study shows an association between higher recently raised ACB and increased acute cardiovascular events. Furthermore, it reveals that protopathic bias can be one of the leading causes by which ACB and cardiovascular events are related since anticholinergic drugs include drugs that might be used to treat early signs of cardiovascular disease [34]. Despite the SCORE2 scale evaluating the probability of having a CVR event in the next ten years, we ignore whether patients in our population had previous cardiovascular events.

The findings above highlight the potential of brief cognitive tests in screening patients with CI. In this sense, 88.9% of patients who failed all three tests were evaluated by a neurologist, showing the usefulness of brief tests in screening for cognitive impairment and the aid it can provide for general practitioners, who often run out of time due to the high burden of care. Moreover, monitoring possible drug interactions and side effects in elderly polymedicated patients by pharmacists, psychologists, general practitioners, and medical specialists is always essential due to the high risk of significant ACB. We have noticed that it is associated with worse SPMSQ tests, but we must not forget that it could be due to the side effects of drugs instead of a cause of dementia. Thus, enhanced communication and coworking between health professionals are vital to give the best attention to patients, and this brief test could be interesting for this patient profile.

Following those mentioned above, general practitioners and cardiovascular healthcare providers may find the MIS interesting since it is very short and may be helpful as a cognitive status follow-up. Both tests are completed in less than 5 min, so psychologists, pharmacists, or primary care doctors could use it for quick and easy monitoring, facilitating referral to a specialist if necessary, especially when CVR and ACB are detected.

As a result, detecting these patients from primary care or other specialties, such as psychiatry or cardiology, can be an opportunity to modify the progression of decline, empowering healthcare professionals and motivating them to take action.

Long-term care pathways, from diagnosis to end of life, for people with dementia are often fragmented. The human rights of people with dementia are frequently denied both in the community and in care homes, underscoring the urgent need for a global action plan for a world where dementia can be prevented and where people with dementia and their caregivers strive to optimize their quality of life. Patients with dementia should receive the care and support they need to reach their potential with dignity, respect, autonomy, and equity, highlighting the importance of this issue.

### Strengths and Limitations

CVR scores primarily predict future cardiovascular events and may not accurately reflect the current cardiovascular health of the patients. Nevertheless, CVR scales such as SCORE2 are easily implemented in electronic medical records. Therefore, this scale could quickly alert doctors about CVR and simultaneously assess the MIS test to monitor patients’ cognitive status from primary healthcare centers. Similarly, including anticholinergic risk scales on medical records programs could have the same effect. We used the CRIDECO scale because it is based on a recent systematic review and demonstrated higher accuracy in predicting CI than the traditional ACB scale [10]. Another strength is that several studies explore the effects of ACB or CVR on cognition. Nevertheless, most of them study either CVR or ACB and do not explore their potential influence on each other or their possible added effect.

In this study, we have focused on each of the evaluated cognitive domains. The aim was to assess whether there is an association between CVR and ACB and the presence of alteration in each cognitive domain, using the test results as a proxy. Failing at least one of them could be a possible sign of cognitive impairment due to the sensitivity and specificity of each test. However, we could not evaluate activities of daily living, which could allow us to understand the patient profile better. We acknowledge that we did not have the final diagnosis of all the patients. However, we have included the information regarding patients who finally obtained an interview with a neurologist to allow a more comprehensive interpretation of the results.

Despite this, we observed in previous studies using these cognitive tests that a significant percentage of patients who fail at least one of these brief cognitive tests are diagnosed with CI when evaluated by a neurologist [15].

On the other hand, we acknowledge a potential selection bias in our study. We included only patients who had the clinical variables of interest necessary to calculate both CVR and ACB. Additionally, we excluded those patients who were not taking any medication (*n* = 2), resulting in a lower sample size with a study population composed of polymedicated patients, possibly with cardiovascular comorbidities. Those mentioned above should be considered when interpreting the results. Still, the selected variables are commonly recorded in individuals over 70 years of age due to the high prevalence of cardiovascular diseases in this population. As such, our study focused on patients around 74 years old. Although this is not a limitation, longitudinal studies controlling CVR and ACB from middle age and introducing biological biomarkers, such as imaging or plasma samples, could enhance understanding of the underlying mechanisms.

Lastly, as a strength, all patients were interviewed in person during their consultation. Thus, pharmacological data are accurate and reflect the medication the patient is taking. Future studies must include more participants and explore different life courses to analyze these associations comprehensively and to delve deeper into factors like sex differences, ethnicity, or race, which we could not explore in our study.

## 5. Conclusions

Controlling risk factors during middle age could promote preventive action against cognitive decline. Administrating brief cognitive tests in routine clinical practice and the potential to select the most accurate test based on individual patient profiles present an opportunity to identify interventions to reduce the likelihood of CI. Specifically, the MIS test benefits patients over 74 with cardiovascular risk. The SPMSQ is also effective for estimating cognitive impairment odds in patients with anticholinergic burden.

## Figures and Tables

**Table 1:** Description of the participants in this study based on the presence of cognitive impairment.

Variable	Measure	CI+ (*n* = 104)	CI− (*n* = 68)	*p*-Value
Age	Mean (sd)	74.61 (8.25)	73.67 (10.54)	0.54
Sex (Female)	*n* (%)	74 (71.15)	42 (61.76)	0.20
Educational level (High)	*n* (%)	22 (21.4)	49 (73.1)	<0.01
Number of concomitant drugs	Mean (sd)	6.77 (3.40)	5.56 (3.05)	0.02
Total anticholinergic burden	Mean (sd)	1.95 (1.72)	1.01 (1.33)	<0.01
Significant anicholinergic burden (CALS > 2)	Yes, *n* (%)	35 (33.65)	9 (13.24)	<0.01
Type 2 Diabetes features				
HbA1c	Mean (sd)	6.68 (1.22)	5.68 (1.12)	<0.01
Glucose	Mean (sd)	105.08 (26.86)	106.58 (26.90)	0.74
Antidiabetic agents intake	Yes, *n* (%)	26 (25.00)	21 (29.41)	0.52
Hypertenssion-related variables				
SBP (mmHg)	Mean (sd)	134.84 (14.85)	129.43 (17.75)	0.04
DBP (mmHg)	Mean (sd)	73.88 (10.63)	75.01 (9.55)	0.44
Antihypertensive drugs intake	Yes, *n* (%)	66 (63.46)	47 (69.12)	0.44
Dyslipidemia-related variables				
Total cholesterol (mg/dL)	Mean (sd)	192.99 (44.22)	190.19 (40.91)	0.67
HDL cholesterol (mg/dL)	Mean (sd)	56.64 (15.43)	57.57 (19.57)	0.74
LDL cholesterol (mg/dL)	Mean (sd)	110.21 (35.04)	106.13 (35.95)	0.46
Lipid-lowering drugs intake	Yes, *n* (%)	49 (60.49)	51 (58.62)	0.80
SCORE2 (Score)	Mean (sd)	13.63 (8.26)	12.65 (7.55)	0.42
SCORE2 (low risk)	*n* (%)	15 (14.42)	12 (17.65)	0.56
SCORE2 (moderate risk)	*n* (%)	53 (50.96)	29 (42.65)	
SCORE2 (high risk)	*n* (%)	36 (34.62)	27 (39.71)	
CVR Alert	Yes (%)	29 (27.88)	22 (32.35)	0.53
MIS	Mean (sd)	4.38 (2.27)	7.28 (0.94)	<0.01
SPMSQ	Mean (sd)	2.91 (1.99)	0.60 (0.74)	<0.01
SVF	Mean (sd)	10.61 (4.36)	19.16 (6.95)	<0.01
Group 1 CVR+, ACB+	Mean (sd)	29 (11.76)	8 (27.88)	0.02
Group 2 CVR+, ACB−	Mean (sd)	48 (70.59)	60 (57.69)	
Group 3 CVR−, ACB+	Mean (sd)	1 (1.47)	6 (5.77)	
Group 4 CVR−, ACB−	Mean (sd)	11(16.18)	9 (8.65)	

Abbreviations: ACB: Anticholinergic burden; CI: Cognitive Impairment; CVR: Cardiovascular risk; HbA1c: Glycated hemoglobin; SBP: Systolic blood pressure; DBP: Diastolic blood pressure; MIS: memory impairment screen and SVF: Semantic Verbal Fluency. Variables with incomplete data (number of patients without data): HbA1c (*n* = 101); glucose (20); Lipid-lowering drugs (*n* = 4).

**Table 2:** Relationship of brief cognitive tests with SCORE2 and CALS scales.

Variable	Measure	Failed MIS (*n* = 60)	Passed MIS (*n* = 112)	*p*-Value	Failed SVF (*n* = 50)	Passed SVF (*n* = 122)	*p*-Value	Failed SPMSQ (*n* = 64)	Passed SPMSQ (*n* = 108)	*p*-Value
SCORE2 (score)	Mean (sd)	15.92 (9.12)	11.81 (6.92)	<0.01	14.78 (8.82)	12.61 (7.55)	0.13	14.15 (8.93)	12.70 (7.35)	0.27
SCORE2 (Low)	*n* (%)	4 (6.67)	23 (20.54)	0.04	5 (10.00)	22 (18.03)	0.36	12 (18.75)	15 (13.89)	0.49
SCORE2 (Moderate)	*n* (%)	29 (48.33)	53 (43.15)		27 (54.00)	55 (45.08)		27 (42.19)	55 (50.93)	
SCORE2 (High)	*n* (%)	27 (45.00)	36 (40.18)		18 (36.00)	45 (36.89)		25(39.06)	38 (35.19)	
CVR Alert (SCORE2)	Yes (%)	56 (93.33)	89 (79.46)	0.02	52 (81.25)	93 (86.11)	0.40	45 (90.00)	100 (81.97)	0.19
Total anticholinergic burden	Mean (sd)	1.75 (1.70)	1.49 (1.60)	0.33	1.74 (1.37)	1.52 (1.74)	0.37	2.03 (1.84)	1.31 (1.45)	<0.01
CALS > 2	*n* (%)	16 (26.67)	28 (25)	0.81	14 (28)	30 (24.59)	0.64	22 (34.38)	22 (20.37)	0.04

Abbreviations: CALS: CRICECO Anticholinergic Burden Scale; CVR: Cardiovascular risk; MIS: Memory impairment screen and SVF: Semantic Verbal Fluency. Cut-off points for probable cognitive decline: MIS ≤ 4 points; SPMSQ ≥ 3 points; SVF < animals.

**Table 3:** Probability of failing the MIS, SVF, and SPMSQ tests according to patient group.

Group	Failed MIS OR (IC)	*p*-Value	Failed SVF OR (IC)	*p*-Value	Failed SPMSQ OR (IC)	*p*-Value
Group 1 CVR+, ACB+	5.47 (1.31, 37.8)	0.04	2.17 (0.63, 8.78)	0.74	1.42 (0.47, 4.39)	0.53
Group 2 CVR+, ACB−	5.73 (1.54, 37.2)	0.02	1.68 (0.56, 6.22)	0.38	0.69 (0.26, 1.90)	0.46
Group 3 CVR−, ACB+	3.60 (0.36, 37.09)	0.25	0.67 (0.03, 5.77)	0.24	2.00 (0.35, 12.6)	0.44

Logistic regressions. Reference level= Group 4 (CVR−, ACB−). Abbreviations: MIS: Memory Impairment Screening; SVF: Semantic Verbal Fluency; SPQMS: Short Portable Mental State Questionnaire by Pfeiffer. Cut-off points for probable cognitive decline: MIS ≤ 4 points; SPMSQ ≥ 3 points; SVF < animals.

## Data Availability

Data are available under request.

## Supplementary Materials

The following supporting information can be downloaded at https://www.mdpi.com/article/10.3390/jcm13196009/s1, Figure S1: Number of failed cognitive tests and scores on SCORE2 (left) and total anticholinergic burden (right); Table S1: Sensitivity, specificity, and duration of brief tests used in the detection of cognitive impairment; Table S2: Patient’s group classification; Table S3: Probability of Failing the MIS, SVF, and SPMSQ Tests According to Patient Group adjusted by educational level.
