# Supplementary material for: Selecting a Brief Cognitive Screening Test Based on Patient Profile: It Is Never Too Early to Start
Source: J Clin Med. 2024 Oct 9;13(19):6009. doi: 10.3390/jcm13196009 (PMC11477581; doi:10.3390/jcm13196009)
Supplement: Supplementary file 1 [file jcm-13-06009-s001.zip › jcm-3210131-supplementary.pdf]

Journal of
Clinical Medicine

MDPI

Supplementary material of "Selecting a Brief Cognitive Screening Test Based on Patient Profile: It Is Never too Early to Start"

Table S1. Sensitivity, specificity, and duration of brief tests used in the detection of cognitive impairment.

Validated screening tests for cognitive impairment	Sensitivity	Specifity	Duration (minutes)
Memory Impairment Screening	0,74	0,96	2
Short Portable Mental State Questionnaire by Pfeiffer	0,85	0,79	3
Semantic Verbal Fluency	0,74	0,80	1

Cut-off points for probable dementia: Memory Impairment Screening: ≤4 points; Short Portable Mental State Questionnaire by Pfeiffer ≥ 3 points; Semantic Verbal Fluency: Recall of < 10 animals.

Table S2. Patient's group classification.

Groups	Significant anticholinergic burden (CALS ≥ 3)	Non- significant anticholinergic burden (CALS < 3)
High CVR (Moderate or high according SCORE2)	Group 1	Group 2
Low CVR (Low according SCORE2)	Group 3	Group 4

Abbreviations: CALS= CRIDECO Anticholinergic Burden Scale; CVR= Cardiovascular Risk; SCORE2= Systematic Coronary Risk Evaluation-2.

Table S3. Probability of Failing the MIS, SVF, and SPMSQ Tests According to Patient Group adjusted by educational level.

Group	Failed MIS OR (IC)	P-value	Failed SVF OR (IC)	P-value	Failed SPMSQ OR (IC)	P-value
Group 1 CVR+, ACB+	3.13 (0.69, 22.41)	0.18	1.30 (0.34, 5.59)	0.71	0.55 (0.14, 2.04)	0.37
Group 2 CVR+, ACB -	4.88 (1.27, 32.25)	0.04	1.36 (0.43, 5.23)	0.62	0.38 (0.12, 1.24)	0.11
Group 3 CVR-, ACB +	1.93 (0.19, 20.50)	0.56	0.32 (0.01, 2.99)	0.37	0.51 (0.08, 3.63)	0.49
Low educational level	3.17 (1.53, 6.88)	<0.01	3.50 (1.63, 7.99)	<0.01	8.92 (3.92, 22.73)	<0.01

Logistic regressions adjusted by educational level. Reference levels= Group 4 (CVR-, ACB -) and high educational level.: MIS: Memory Impairment Screening; SVF: Semantic Verbal Fluency; SPQMS: Short Portable Mental State Questionnaire by Pfeiffer. Cut-off points for probable cognitive decline: MIS ≤4 points; SPMSQ ≥ 3 points; SVF < animals.

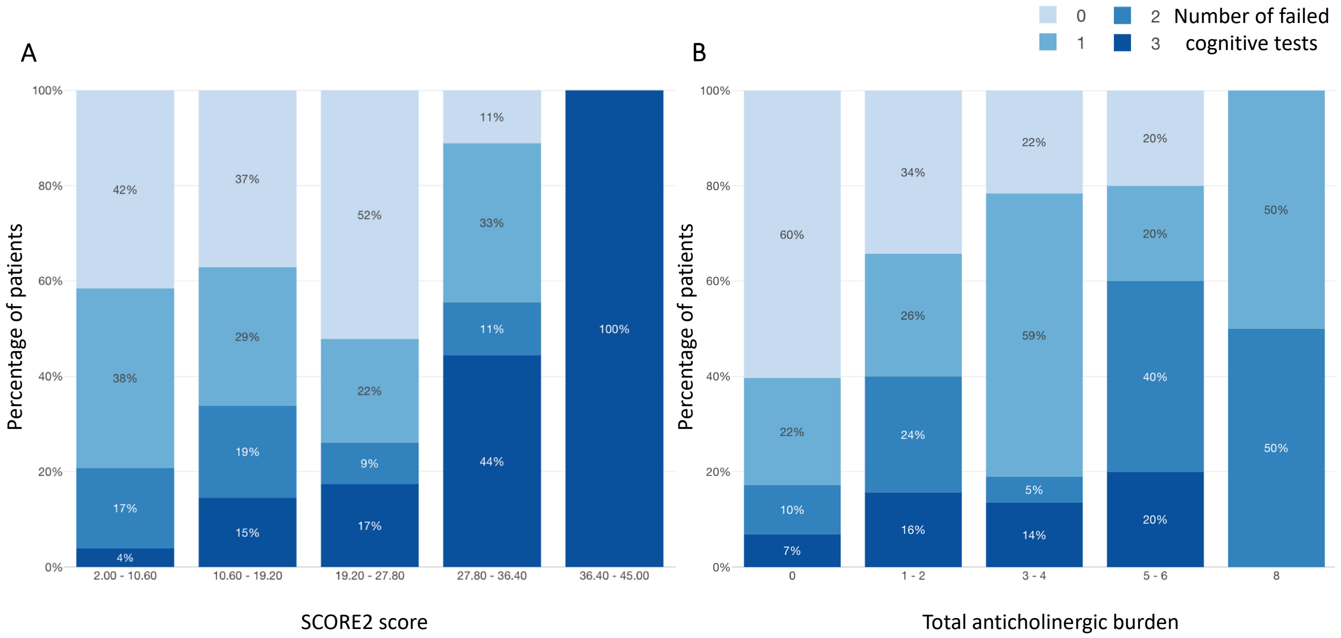

Figure S1. Number of failed cognitive tests and scores on SCORE2 (left) and total anticholinergic burden (right).
